# COVID-19 pandemic vaccination strategies of early 2021 based on behavioral differences between residents of Tokyo and Osaka, Japan

**DOI:** 10.1186/s13690-022-00933-z

**Published:** 2022-08-04

**Authors:** Hidenori Yasuda, Fuyu Ito, Ken-ichi Hanaki, Kazuo Suzuki

**Affiliations:** 1 Department of Mathematics, Faculty of Science, Josai University, Hirakawa-cho, Chiyoda-ku, Tokyo, 102−0093 Japan; 2 Asia International Institute of Infectious Disease Control, Teikyo University, Kaga 2-11-1 Itabashi-ku, Tokyo, 173-8605 Japan; 3 Management Department of Biosafety, Laboratory Animal, and Pathogen Bank, National Institute of Infectious Diseases, Shinjuku-ku, Tokyo, 162-8640 Japan; 4 Japan Infection Control Association, Ogura-cho 40-3 Kitashirakawa Sakyo-ku, Kyoto, 606-8264 Japan; 5 Research Institute of Disaster Medicine, Chiba University, Inohana 1-8-1, Chuo-ku, Chiba, 260-8670 Japan

**Keywords:** Age-specific behavior, Big cities, COVID-19, Simulation, Vaccination

## Abstract

**Background:**

During the fourth COVID-19 wave in Japan, marked differences became apparent in the scale of the epidemic between metropolitan Tokyo in eastern Japan and Osaka prefecture in western Japan.

**Methods:**

Public epidemic data were analyzed, with performance of mathematical simulations using simplified SEIR models.

**Results:**

The increase in the number of infected persons per 100,000 population during the fourth wave of expansion was greater in Osaka than in Tokyo. The basic reproduction number in Osaka was greater than in Tokyo. Particularly, the number of infected people in their 20 s increased during the fourth wave: The generation-specific reproduction number for people in their 20 s was higher than for people of other generations. Both Tokyo and Osaka were found to have strong correlation between the increase in the number of infected people and the average number of people using the main downtown stations at night. Simulations showed vaccination of people in their 60 s and older reduced the number of infected people among the high-risk elderly population in the fourth wave. However, age-specific vaccination of people in their 20 s reduced the number of infected people more than vaccination of people in their 60 s and older.

**Conclusions:**

Differences in the epidemic between Tokyo and Osaka are explainable by different behaviors of the most socially active generation. When vaccine supplies are adequate, priority should be assigned to high-risk older adults, but if vaccine supplies are scarce, simulation results suggest consideration of vaccinating specific groups among whom the epidemic is spreading rapidly.

## Background

In Japan, coronavirus disease 2019 (COVID-19) began to spread in January 2020. Several epidemic waves have occurred since then. As the epidemic began to expand throughout the population, the Japanese government declared a state of emergency, asking people to adjust and limit normal daily activities [1]. These limitations included refraining from going out, moving around unnecessarily, and traveling to other areas. Furthermore, the recommended maximum number of people at events was capped at 5,000 and 50% capacity. Restaurants serving alcoholic beverages and providing karaoke facilities were asked to close. Other restaurants were advised to close at 8 p.m. Telecommuting was implemented widely. Although these measures were not legally binding, the epidemic was brought under control after declaration of the state of emergency [2, 3].

In Japan, COVID-19 is widespread among people in their 20 s. It can cause severe illness and disability in elderly people. Globally, COVID-19 has age-specific effects. Typically, the COVID-19 pandemic has produced a markedly low proportion of cases among children, with children having lower susceptibility to infection and a lower propensity to exhibit clinical symptoms [4]. The susceptibility of children under 10 years of age to COVID-19 infection is significantly lower than that of adolescents and middle-aged people, whereas the susceptibility of people aged 60 years and older is higher [5]. After the age of 60 years, people infected with severe acute respiratory syndrome coronavirus 2 (SARS-CoV-2) are divisible into two groups based on symptoms [6]; the mortality rate increases sharply after the age of 65 years [7]. From a survey of 45 countries in Africa, Asia, Europe, North America, and Latin America, the mortality rate between the ages of 30 and 65 years showed a log-linear dependence on age, but regional differences were apparent after the age of 65 years: social contact is less frequent in that group [8].

During the fourth wave in Japan, which began in March 2021, a large difference was found in the number of infected people between Metropolitan Tokyo (Tokyo) and Osaka prefecture (Osaka). This difference was not apparent in data of the third wave beginning in January 2021. In Osaka, the increase in infections among people in their 20 s was pronounced. Younger people in Osaka went out more often at night than people in Tokyo [9, 10].

For this study, the scale of the epidemic between Tokyo and Osaka during the fourth wave of COVID-19 was compared by age group, specifically using simulations to ascertain whether the difference is attributable to behavioral factors. Effects of prioritization of vaccination for people of certain generations to control the epidemic were assessed using mathematical models.

## Methods

### Analysis of epidemic data

The numbers of people infected with COVID-19 were referred from weekly data published by the Tokyo Metropolitan Government and Osaka Prefectural Government. The Tokyo Metropolitan Government publishes data of positive tests on the web, in addition to the decadal generation and gender of infected people [2]. The Osaka Prefectural Government publishes the number of infected people, along with their decadal generation, in daily press releases [3]. Next, we quantified the number of people at major train stations in Tokyo and Osaka at night based on regional economic data provided by Japanese government agencies [9, 10].

### Mathematical simulation

**Fig. 1:**
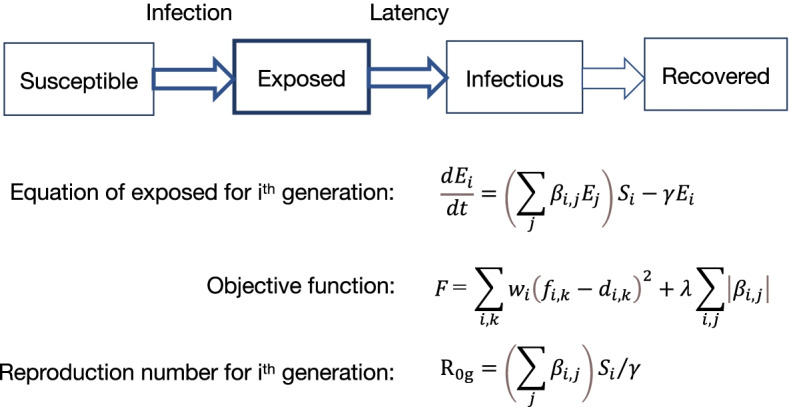
Schematic showing the simplified model

For the simulation, a simplified susceptible (S), exposed (E – infected but not infectious), infectious (I), and recovered (R) (SEIR) compartment model was constructed by dividing the population in the incubation period into groups of under 10 years old, 10 s, 20 s, 30 s, 40 s, 50 s, and 60 and more years old. The numbers of infected people in Tokyo and Osaka were small compared to the total population in those areas. Therefore, changes in the susceptible population were ignored. Legally categorized as a Category II Infectious Disease in Japan, COVID-19 is treated equivalently to high-pathogenic avian influenza A/H5N1. Infectious patients are legally obligated to be admitted to a designated medical facility. If symptoms are sufficiently minor that they do not require hospitalization, or if asymptomatic but PCR tests are positive, then the patients are quarantined at a hotel that was specially rented entirely by the local government at the time of the fourth wave. Because of their quarantine from the community, infectious persons are not included in the community epidemic model. Infection was able to spread during the incubation period. Therefore, only the E (exposed) element of the SEIR model was computed, assuming that infection was transmitted among the susceptible population during the incubation period after exposure. The simplified model equations and objective function *F* are presented in Fig. 1.

Here, *E* represents the population during the incubation period, *S* stands for the susceptible population, β is the transmission coefficient, and γ denotes the isolation rate. Subscripts *i* and *j* denote generations, *k* signifies weeks, *f* and *d* respectively represent calculated and data values, *w* signifies the generation weight, and λ is the weight of the penalty term. The model parameters are *S*, β, and γ. The susceptible population *S* is taken from census data of Tokyo and Osaka. Parameter γ is the reciprocal of the incubation period. Estimated values are used for β. For the model, the incubation period was set as 1 week, based on earlier reported incubation periods of 5.6–7.7 days [11] (median = 5.1 days [12]; mean = 4.82 days [13]). The transmission coefficient for infection was obtained from data by fitting an objective function with a penalty term to the data, which prevents overfitting [14]. The generation weight was set as 10 for Osaka residents in their 60 s and older during the “third wave convergence period”, and 1 for the rest. Because differences in the data among the 30 s, 40 s and 50 s generations became smaller during the “third wave convergence period” in Osaka (Fig. 4.b), some risk of contamination among data exists in the calculation of the minimization of the objective function. Therefore, we weighted the data of people 60 and older to control error in the calculations. The weight of λ was set to ca. 10^–6^ of the error terms in the sum of squares. The generation-specific reproduction number of the simplified model for the *i*^th^ generation was calculated using *R*_0*g*_ in Fig. 1. The basic reproduction number of the simplified model *R*_0_ was calculated as the average generation-specific reproduction number. When the number of generations is 1 (conventional SEIR model), the solution of the simplified model becomes an exponential function. Therefore, it is applicable to the early stage of epidemic expansion and the “epidemic convergence period.” However, the simplified model is robust because it reflects features of the spread of epidemics. It is therefore applicable to simulation in suitable situations. Because of the emergence of variant strains, people's behavior changed and varied over time during declarations of states of emergency for COVID-19. During the third wave, the strain B.1.1.214, which was presumed to have mutated in Japan, was the predominant strain from October 2020, whereas most strains of the fourth wave were alpha strains VOC-202012/01 (lineage B.1.1.7); other strains were limited [15, 16]. In addition, a coercive state of emergency was issued during the “third wave convergence period”. However, no enforceable declarations were issued during the fourth wave expansion period. People's behaviors changed dramatically after the declaration of the state of emergency.

## Results

### Numbers of infected people

**Fig. 2:**
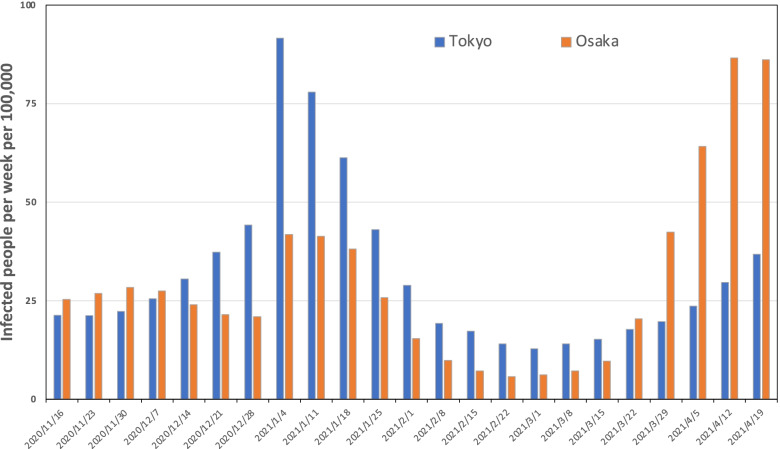
Number of infected people in Tokyo and Osaka per week per 100,000 population

Figure 2 portrays the numbers of infected people in Tokyo and Osaka during the convergence period of the third wave of the epidemic in Japan and the expansion period of the fourth wave. The number of infected people per 100,000 population was aggregated weekly for each generation, from the week beginning November 16, 2020 to the week beginning April 19, 2021. The third wave peaked during the New Year holidays and converged after the second declaration of emergency was issued at the beginning of 2021. During the fourth wave, in the week of March 18, there were more infected people in Tokyo than in Osaka. However, during the week of March 22, the number of infected people in Osaka exceeded that in Tokyo: The scale of the epidemic became greater than that in Tokyo.

### Proportions of infected people

**Fig. 3:**
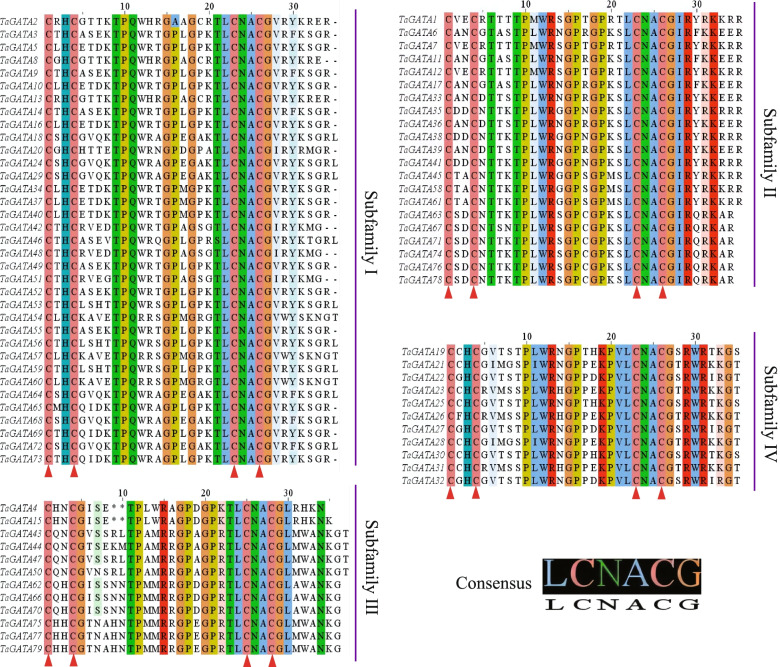
Percentage of infected people by generation: (**a**) Tokyo and (**b**) Osaka. The u20 denotes under 20 years of age and the o60 denotes over 60 years of age

The proportions of infected people in Tokyo and Osaka were analyzed by decadal generation. The decadal generation categories were under 20 years, 20 s, 30 s, 40 s, 50 s, and 60 s and older. Figure 3a and b show percentages of infected people in Tokyo and Osaka by decadal generation. The proportion of infected people in their 60 s and older increased during the third wave convergence period in January 2021, whereas the proportion of infected people in their 20 s increased during the fourth wave expansion period in March and April 2021.

### Third wave convergence period

**Fig. 4:**
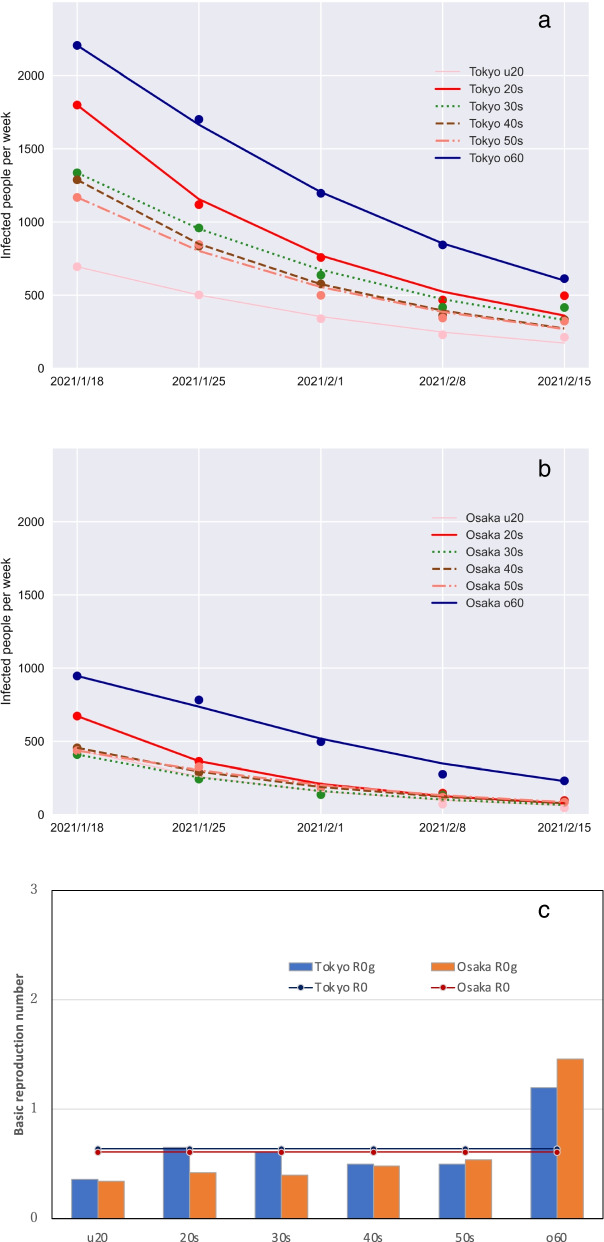
Third wave of the COVID-19 epidemic in Tokyo and Osaka. Infected people in (**a**) Tokyo **b**) and Osaka, and (**c**) the basic reproduction number (bar) and generation-specific basic reproduction number. The u20 denotes under 20 years of age and the o60 denotes over 60 years of age

Parameters were fit to the simulation model using data for the number of infected people during the convergence period of the third wave. Simulation results were obtained for the model fitted with the number of infected people during January 8 – February 15, 2021. The results obtained for Tokyo are portrayed in Fig. 4a. Those for Osaka are portrayed in Fig. 4b. Figure 4c shows the basic and generation-specific reproduction numbers of the model for the third wave convergence period. In both Tokyo and Osaka, the basic reproduction number was about 0.6, but the generation-specific reproduction number exceeded 1.0 for the generation in their 60 s and older.

### Fourth wave expansion period

**Fig. 5:**
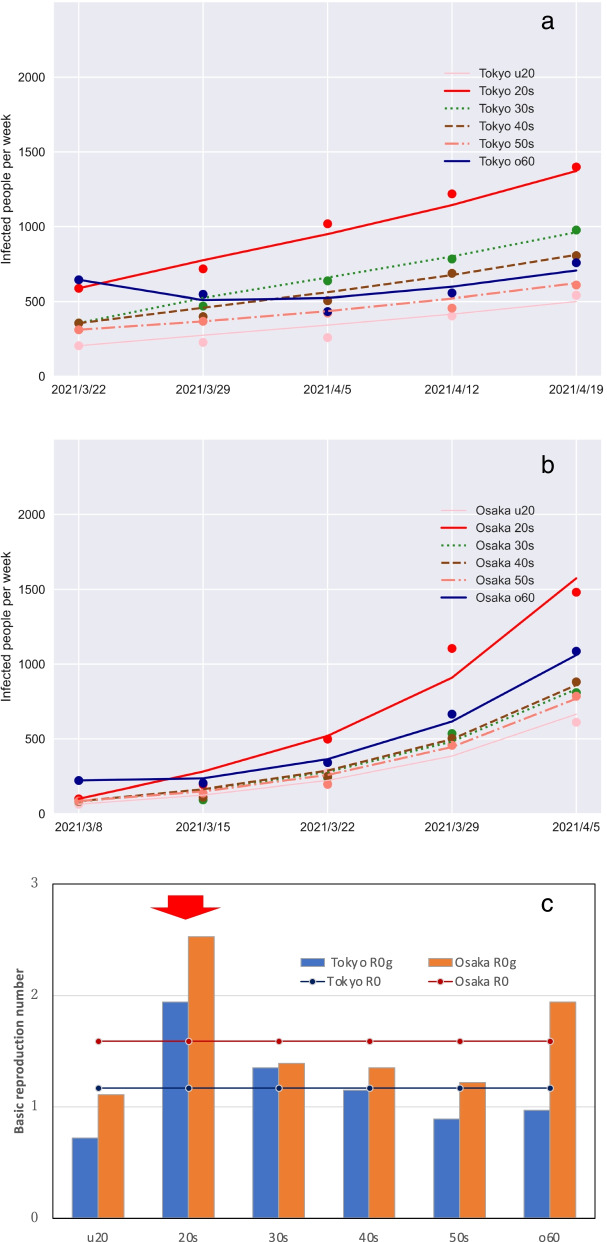
Fourth wave of the COVID-19 epidemic in Tokyo and Osaka. Infected people in (**a**) Tokyo and (**b**) Osaka, and (**c**) the basic reproduction number (bar) and generation-specific basic reproduction number. The u20 denotes under 20 years of age and the o60 denotes over 60 years of age

The results of parameter fitting for the fourth wave expansion period are presented in Figs. 5a and b. For Tokyo, the data for the weeks of March 22 – April 19, 2021 were used for fitting, whereas the data for Osaka were for the weeks of March 8 – April 5, 2021. The restrictions associated with the second emergency declaration in Osaka were lifted 2 weeks earlier in Osaka than in Tokyo. The fourth wave onset occurred 2 weeks earlier than in Tokyo. Figure 5c presents the basic reproduction number for the fourth wave expansion period and the generation-specific reproduction number in the model simulations. The larger scale of the epidemic in Osaka than Tokyo is reflected in the fact that the number of basic reproducers in Osaka was greater than in Tokyo. In both Tokyo and Osaka, the generation-specific reproduction number for people in their 20 s was greater than that of other generations, as shown by arrows in the figure. In Osaka, the generation-specific reproduction number of people in their 60 s and older was also large.

### Effects of people’s behavior

**Fig. 6:**
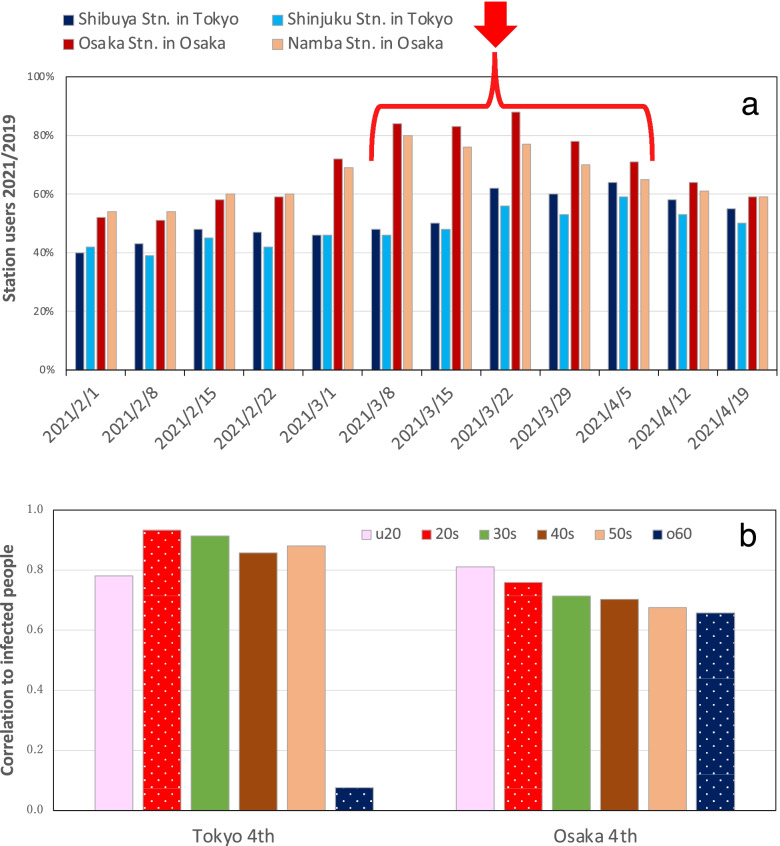
Number of people at major train stations during 20:00 – 24:00 compared to 2019. **a** Shibuya and Shinjuku stations in Tokyo, and Osaka and Namba stations in Osaka. **b** Correlation between number of people and number of infected people during expansion of the fourth wave. The u20 denotes under 20 years of age and the o60 denotes over 60 years of age

To assess the effects of people’s behavior on the spread of the COVID-19 pandemic, we obtained data for people using major train stations in Tokyo and Osaka. We compared the numbers of station users during 20:00 – 24:00 at Shibuya and Shinjuku stations in Tokyo, and at Osaka and Namba stations in Osaka, with their respective numbers during the same week in 2019 (Fig. 6a). All stations are in downtown areas. The numbers of users near the major stations were smaller than in 2019 because people were asked to refrain from going out during the state of emergency, although no lockdown was implemented in Japan. However, the numbers of user populations at both the Osaka and Namba stations in Osaka were considerably larger in mid-March of 2021, after the end of the restrictions associated with the second emergency declaration, compared to the week prior, as shown by the mark under the arrow in Fig. 6a. Figure 6b shows the correlation coefficient between the increase in number of infected people by decadal generation and the number of users major stations during the prior week. Strong correlation was found between the average numbers of users at the two major stations in both Tokyo and Osaka during 20:00 – 24:00 and the increase in number of infected people, except for people in their 60 s and older in Tokyo.

### Age-specific vaccination

**Fig. 7:**
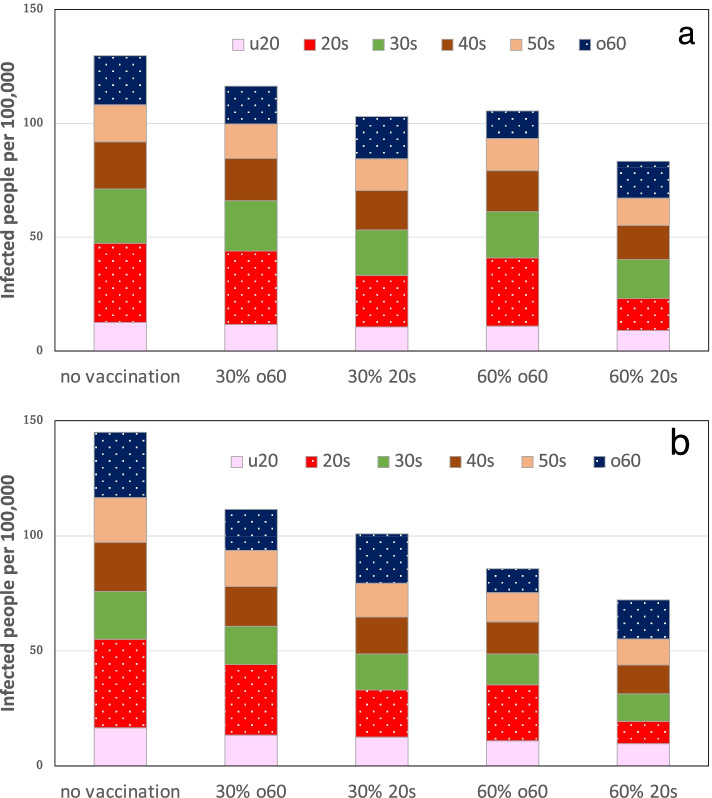
Simulated numbers of infected people per 100,000 population during the fourth wave. 30% or 60% of the population aged 60 and older vaccinated, 20 s vaccinated, (**a**) Tokyo, (**b**) Osaka. The u20 denotes under 20 years of age and the o60 denotes over 60 years of age

In Japan, medical personnel and people aged 65 years and older were vaccinated in April 2021. However, vaccinations were not fully implemented until mid-May, after the peak of the fourth wave had passed. For the fourth wave of expansion, we simulated the reduction in the number of infected people when age-specific vaccination was implemented. The target population for vaccination was high-risk people older than 60 years old and people in their 20 s, who were the most frequently infected. For the simulation, vaccinated people were excluded from the susceptible population. Figure 7a and b show the numbers of infected people from March 22 through April 19, 2021, in Tokyo and from March 8 through April 5, 2021 in Osaka. For both cities, it was assumed that 30% and 60% of the population over the age of 60 and in their 20 s, respectively, had been vaccinated. If the vaccination rates for people in their 20 s and 60 s and older had been the same, then vaccination in the 60 s and older was shown to reduce the number of infected high-risk elderly people. However, vaccination of people in their 20 s reduced the number of infected people more than vaccination of people in their 60 s and older. In addition, the decrease in the proportion of infected people because of vaccination was greater in Osaka than in Tokyo. In the calculations presented above, vaccine effectiveness was assumed to be 100%. The first report of vaccine effectiveness in Japan was based on a survey conducted during June–July 2021, when the alpha strain B.1.1.7 was replaced by the delta strain B.1.617.2; the effectiveness was 95% (95% CL 72–100) [17]. If the effectiveness was 72%, if 60% of the population in their 20 s, and 30% of the population 60 years of age and older were vaccinated, the numbers of infected persons per 10,000 persons would be 93.5 and 120.4 in Tokyo, respectively. In Osaka, the numbers were 86.8 and 120.0, respectively. The differences in the numbers of infected persons aged 60 years or older were about 2% in Tokyo and about 6% in Osaka.

## Discussion

This study used a simplified SEIR model to evaluate reproduction numbers. The basic reproduction number obtained from the model simulations was about 0.6 in both Tokyo and Osaka during the third wave convergence period, whereas it was 1.17 in Tokyo and 1.59 in Osaka during the fourth wave expansion period. The epidemic expansion period was short because of the state of emergency. For that reason, the data were scarce, leading to overfitting. Overfitted models are unsuitable for predicting epidemics because they reproduce the peculiar characteristics of the data with which they are fitted [18]. During early expansion or convergence phase of an epidemic, the data show a common pattern. Data-specific fluctuations that cause overfitting are small. In addition, overfitting is suppressed by an objective function with a penalty term. The model deals with the COVID-19 epidemic expansion within megacities because the movement of people over a wide area has been reduced greatly as a result of public requests for self-restraint from travel across prefectures in Japan. The COVID-19 epidemic shows complex patterns in megacities. From April through June 2020, the 23 wards of Tokyo were divisible into three zones according to COVID-19 epidemic patterns: downtown, within the circumference of the Japan Railway Yamanote Line, and outer residential areas [19]. For influenza, detailed simulations of Tokyo have been conducted using agent models to model people’s daily behavior [20, 21]. However, the necessary data for agent models, such as infection rates in homes, workplaces, restaurants, and commuter trains, are not yet available for COVID-19.

The effective reproduction numbers for Japan were reported as 0.6–0.8 in mid-February 2021 and as 1.1–1.3 at the end of March [22]. On March 22, 2021, the effective reproduction number reported for Osaka was 1.74 [23], which resulted from the rapid spread of infection because of an increase in people’s social activities from the middle of March [23]. The generation-specific reproduction numbers of people in their 20 s were 1.94 and 2.53 in Tokyo and Osaka, respectively, during the fourth wave, which were higher than for other generations. During both the Tokyo and Osaka epidemics, the proportion of infected people in their 20 s increased during the expansion stage, whereas the proportion of infected people in their 60 s and older increased during the convergence period. In the United States (US), the reported reproduction number for people under 20 years old was about half that of people in their 20 s. Moreover, during the expansion stage of the epidemic, most cases were found among the 20–59-year age group. By contrast, after the peak of the epidemic, the numbers of infected people aged under 20 and over 60 years increased [4].

In Japan, the proportion of infected people in their 20 s increased during the expansion period, indicating that this age group played a major role in the epidemic. The fourth wave was larger in Osaka than in Tokyo, as was the spread of infection among people in their 20 s. The generation-specific reproduction number for people in their 20 s was 1.94 in Tokyo and 2.53 in Osaka. When one specifically examines the night users of major stations in downtown areas of Tokyo and Osaka to ascertain differences in behavioral changes in people in their 20 s, the numbers of users at Shibuya and Shinjuku stations in Tokyo remained fundamentally constant, at about 40% of the 2019 population from early February to mid-March, with a gradual increase starting from the week of March 22. The station users at the Osaka and Namba stations in Osaka were nearly 60% of the 2019 station users in mid-February; they reached about 80% in March. According to a survey using mobile phones, about 40% of the people present at 21:00 in the period January 4–17, 2021, around Shinjuku and Shibuya stations were in their 20 s [24]. In addition, the downtown area was regarded as an infected area. The emergency declaration requested that restaurants with alcohol refrain from operating. A survey in Toyama, a regional city, showed that snack and karaoke bars each accounted for more than 20% of infected areas [25]. In Japan, as of April 20, 2021, the nationwide trend of infection spread during the fourth wave was driven mainly by people in their 20 s and 30 s [26]. By comparison, in the US, the contact rate during the epidemic was significantly higher among men younger than 45 years than in the remainder of the population [27]. The epidemic persisted mainly because of 20–49-year-old people [28].

Vaccination against SARS-CoV-2 is necessary to suppress COVID-19, but vaccination in Japan was not done sufficiently in time to prevent or mitigate the fourth wave. How would the outcomes of the epidemic have differed if vaccination had been accomplished in time? Because the epidemic is age-specific, the following two ideas have been proposed. The first is that the epidemic is sustained by young and middle-aged adults. Therefore, vaccination of this generation would best control COVID-19 [9]. The second idea is that vaccination of high-risk older adults represents the best strategy to reduce the burdens of COVID-19 [29]. Studies in the US have demonstrated that vaccinating the elderly population specifically, rather than vaccinating a given group with numerous infected people, was superior in terms of protecting elderly people [29, 30]. In Japan, as in the US, the risk of COVID-19 infection was higher among elderly people [31]; elderly people were assigned priority for vaccination. Simulations using a simplified model of the fourth expansion wave showed that if vaccination rates were equal, the number of infected people in their 60 s and older would be lower in Japan once people in this group had been vaccinated, as in the US. When vaccine supplies are sufficient, priority should be assigned to high-risk elderly people. However, if vaccine supplies are insufficient, the idea of vaccinating a given group with numerous infected people must be considered. The numbers of people in their 60 s and older are 2.2 and 2.9 times greater, respectively, than those of people in their 20 s in Tokyo and Osaka. The amount of vaccine sufficient to vaccinate 30% of people in their 60 s and older would be sufficient to vaccinate 60% of people in their 20 s. The simulation results indicate that vaccinating 60% of people in their 20 s would reduce the total number of infected people more than vaccinating 30% of people in their 60 s and older, although the number of infected people aged over 60 years would be about the same. However, vaccination of people of younger generations has the social benefit of mitigating silent transmission [26], although vaccination of infected people who are less likely to show symptoms might not be achievable in a voluntary manner. In a survey administered before vaccination began, among corporate workers in their 20 s or younger in Japan, 49% intended to be vaccinated; 23% did not [32]. After a vaccine scare, it is difficult to achieve adequate coverage via voluntary vaccination based on a general discussion of the personal benefits and shortcomings of vaccination [33]. There are limitations of this study using the simplified model. The transmission coefficient is a function of time during which there is a change in people's behavior, with or without enforcement measures. In addition, when the number of undetected infections increases, the model must include infectious persons. Furthermore, because the model is spatially uniform, it cannot account for the complex patterns of epidemics within megacities such as Tokyo and Osaka, as discussed above. These discrepancies between the real epidemic and the model must be noted carefully.

## Conclusion

Analyses of epidemic data and results of mathematical simulations indicated that differences in the scale of the epidemic between Tokyo and Osaka are explainable by differences in the behavior of the most socially active generation, mainly people in their 20 s. If vaccine supplies are adequate, then high-risk elderly people should be vaccinated preferentially. However, if vaccine supplies are inadequate, then simulation results suggest that it is worth considering vaccination of specific groups among whom the epidemic is spreading.

## Data Availability

Not applicable.

## Abbreviations

COVID-19: Coronavirus infectious disease in 2019
SARS-CoV-2: Severe acute respiratory syndrome coronavirus 2
SEIR model: Susceptible, exposed, infectious, and recovered compartment model
US: United States
