# Correction: COVID-19 pandemic vaccination strategies of early 2021 based on behavioral differences between residents of Tokyo and Osaka, Japan

**DOI:** 10.1186/s13690-022-00961-9

**Published:** 2022-09-06

**Authors:** Hidenori Yasuda, Fuyu Ito, Ken-ichi Hanaki, Kazuo Suzuki

**Affiliations:** 1 Department of Mathematics, Faculty of Science, Josai University, Hirakawa-cho, Chiyoda-ku, Tokyo, 102−0093 Japan; 2 Asia International Insti- tute of Infectious Disease Control, Teikyo University, Kaga 2-11-1 Itabashi-ku, Tokyo, 173-8605 Japan; 3 Management Department of Biosafety, Labora- tory Animal, and Pathogen Bank, National Institute of Infectious Diseases, Shinjuku-ku, Tokyo, 162-8640 Japan; 4 Japan Infection Control Association, Ogura-cho 40-3 Kitashirakawa Sakyo-ku, Kyoto, 606-8264 Japan; 5 Research Institute of Disaster Medicine, Chiba University, Inohana 1-8-1, Chuo-ku, Chiba, 260-8670 Japan

**Correction: Arch Public Health 80, 180 (2022)**

**https://doi.org/10.1186/s13690-022-00933-z**

Following publication of the original article [1], the authors reported Fig. 3 was the wrong figure from another article due to a typesetting error.

**Fig. 3:**
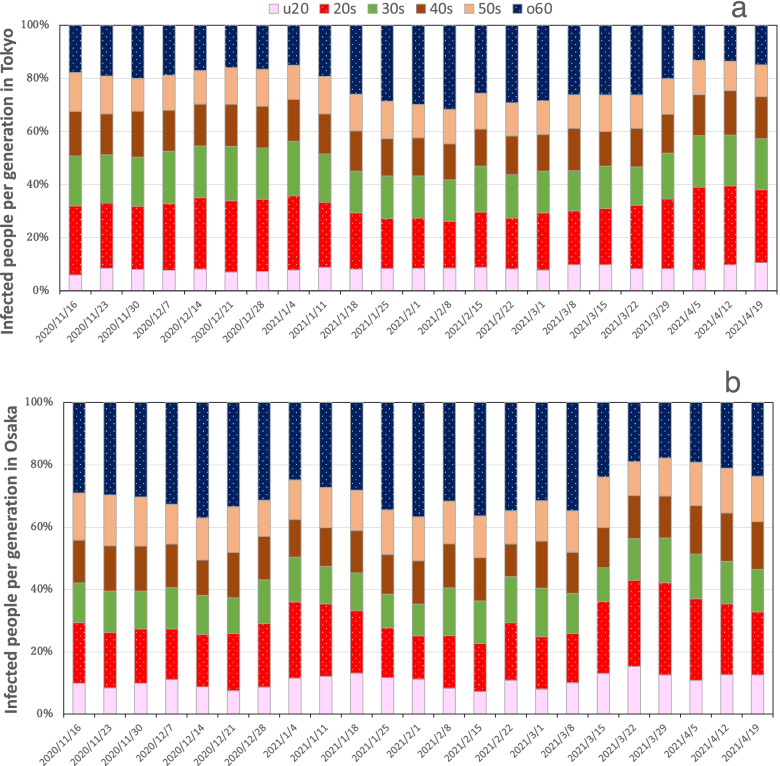
Percentage of infected people by generation: (**a**) Tokyo and (**b**) Osaka. The u20 denotes under 20 years of age and the o60 denotes over 60 years of age

The correct Fig. 3 has been provided in this Correction.

The original article [1] has been corrected.
